# Association of immediate reinsertion of new catheters with subsequent mortality among patients with suspected catheter infection: a cohort study

**DOI:** 10.1186/s13613-022-01014-8

**Published:** 2022-05-07

**Authors:** Yiyue Zhong, Liehua Deng, Limin Zhou, Shaoling Liao, Liqun Yue, Shi Wu Wen, Rihua Xie, Yuezhen Lu, Liangqing Zhang, Jing Tang, Jiayuan Wu

**Affiliations:** 1 Department of Operating Room, Affiliated Hospital of Guangdong Medical University, No.57 People Avenue South, Zhanjiang, 524001 Guangdong China; 2 Department of Critical Care Medicine, Affiliated Hospital of Guangdong Medical University, No. 57, People Avenue South, Zhanjiang, 524001 Guangdong China; 3 Department of Nursing Research, Affiliated Hospital of Guangdong Medical University, No. 57, People Avenue South, Zhanjiang, 524001 Guangdong China; 4 Ottawa Hospital Research Institute Clinical Epidemiology Program, and School of Epidemiology and Public Health, University of Ottawa Faculty of Medicine, 501 Smyth Road, Ottawa, ON K1H 8L6 Canada; 5 The Seventh Affiliated Hospital, Southern Medical University, Foshan, 528200 Guangdong China; 6 Department of Anaesthesiology, Affiliated Hospital of Guangdong Medical University, No.57 People Avenue South, Zhanjiang, 524001 Guangdong China; 7 Department of Clinical Research, Clinical Research Service Center, Collaborative Innovation Engineering Technology Research Center of Clinical Medical Big Data Cloud Service in Medical Consortium of West Guangdong Province, Affiliated Hospital of Guangdong Medical University, No.57 People Avenue South, Zhanjiang, 524001 Guangdong China

**Keywords:** Central venous catheters, Catheter-related bloodstream infection, Reinsertion catheter, Complication, Mortality, Propensity score matching

## Abstract

**Background:**

Central venous catheter (CVC) insertion complications are a prevalent and important problem in the intensive care unit (ICU), and source control by immediate catheter removal is considered urgent in patients with septic shock suspected to be caused by catheter-related bloodstream infection (CRBSI). We sought to determine the impact of immediate reinsertion of a new catheter (IRINC) on mortality among patients after CVC removal for suspected CRBSI.

**Methods:**

A propensity score-matched cohort of patients with suspected CRBSI who underwent IRINC or no IRINC in a 32-bed ICU in a university hospital in China from January 2009 through April 2021. Catheter tip culture and clinical symptoms were used to identify patients with suspected CRBSI. The Kaplan–Meier method was used to analyse 30-day mortality before and after propensity score matching, and adjusted hazard ratios (HRs) and 95% confidence intervals (CIs) for mortality in the matched cohort were estimated with Cox proportional hazards models.

**Results:**

In total, 1,238 patients who had a CVC removed due to suspected CRBSI were identified. Among these patients, 877 (70.8%) underwent IRINC, and 361 (29.2%) did not. Among 682 propensity score-matched patients, IRINC was associated with an increased risk of 30-day mortality (HR, 1.481; 95% CI, 1.028 to 2.134) after multivariable, multilevel adjustment. Kaplan–Meier analysis found that IRINC was associated with the risk of mortality both before matching (*P* = 0.00096) and after matching (*P* = 0.018). A competing risk analysis confirmed the results of the propensity score-matched analysis. The attributable risk associated with bloodstream infection was not significantly different (HR, 1.081; 95% CI 0.964 to 1.213) among patients with suspected CRBSI in terms of 30-day mortality compared with that associated with other infections.

**Conclusions:**

In this cohort study, IRINC was associated with higher 30-day mortality compared to delayed CVC or no CVC among patients with suspected CRBSI. A large-sample randomized controlled trial is needed to define the best management for CVC in cases of suspected CRBSI because IRINC may also be associated with noninfectious complications.

*Trial registration* This study was registered with the China Clinical Trials Registry (URL: http://www.chictr.org.cn/index.aspx) under the following registration number: ChiCTR1900022175.

**Supplementary Information:**

The online version contains supplementary material available at 10.1186/s13613-022-01014-8.

## Background

Central venous catheter (CVC)-associated complications are a prevalent and significant problem in the intensive care unit (ICU) [1]. Catheter-related bloodstream infection (CRBSI) is considered the leading cause of morbidity and mortality in patients with CVCs [2, 3], but CRBSI contributes only modestly and has a better prognosis than other ICU-acquired infections in terms of overall mortality [4–7]. Prompt catheter removal with delayed placement of a new catheter is recommended by the Infectious Diseases Society of America (IDSA) in patients with CRBSI [8], and an expert statement suggests that immediate removal of suspected intravascular catheters is always urgent as a means of source control in patients with septic shock [9]. However, among options that include CVC replacement using a guidewire, insertion of a new CVC, and watchful waiting, the optimal strategy for the management of patients with suspected but unconfirmed CRBSI remains unclear [10]. In clinical practice, the diagnosis of CRBSI is challenging until microbiological culture results are available [10]. A common management strategy for CRBSI is prompt catheter removal followed by immediate reinsertion of a new catheter (IRINC), which prevents interruption of treatments because CVCs provide important access for medical and fluid therapy in critically ill patients [11], especially those who need vasoconstrictive agents [12]. However, catheter insertion increases the risk of complications, including mechanical complications [13], deep vein thrombosis [13, 14], and secondary infections, which are associated with subsequent mortality [4]. Therefore, the decision of whether to remove and reinsert CVCs in critically ill patients with suspected CRBSI has been a debated issue in the management of ICU patients [5, 8, 10, 15], in whom suspected and confirmed CRBSI are one entity and initial management is usually identical. To date, only a few studies have directly or indirectly compared the benefit and harm between reinsertion and no reinsertion in patients whose CVCs have been removed [16–18]. For example, an observational study of 60 cancer patients reported that catheter removal and reinsertion were associated with a moderate to severe symptom burden [16]. A randomized trial showed that mortality did not differ between the catheter removal group and the watchful waiting group in 64 patients with suspected CRBSI [17]. Another randomized controlled trial showed that among 52 patients with suspected CRBSI, the mortality rate in patients who underwent immediate reinsertion and delayed reinsertion of new catheters was not different [18]. Studies with small sample sizes cannot provide strong evidence regarding the association between CVC reinsertion and mortality. Considering the need to maximize benefits in the complex situation of clinical practice, the consequences of a missed catheter-related infection for patients with suspected CRBSI were thought to be more important than the risk of unnecessary catheter removal [17]. Our previous retrospective cohort study showed that catheter removal and IRINC may be associated with 30-day mortality in suspected CRBSI [7]; however, a more comprehensive assessment is needed because it did not meet clinical significance. Therefore, by consensus, we hypothesized that IRINC reduces mortality in patients with suspected CRBSI. In this better-executed cohort study with a larger sample size, we sought to determine the impact of IRINC on 30-day mortality after CVC removal for suspected CRBSI.

## Methods

### Study design and study population

We conducted an investigator-initiated, single-centre, propensity score-matched cohort study of suspected CRBSI patients who underwent IRINC or no IRINC in a 32-bed ICU in China during the period from January 2009 through April 2021. The study was approved by the institutional review board (IRB No. PJ2018-066), conformed to the Official Regulation of Medical Records Management in Medical Institutions with regard to patient data integrity and the principles of the Declaration of Helsinki and was registered in the China Clinical Trials Registry (ChiCTR1900022175). With an index system for institutional electronic laboratory databases, we screened all patients with catheter tip culture results to identify patients with suspected CRBSI, and we confirmed the eligible cases of suspected CRBSI by reviewing the information recorded in the progress notes in the medical charts [5, 8, 10]. The inclusion criteria were age ≥ 18 years, CVC and suspected CRBSI with removal of the catheter. The exclusion criteria were dialysis catheters, peripheral catheterization, length of stay less than 48 h, and lack of accessible medical records.

Data for eligible patients were extracted from paper medical documents, electronic medical records, and electronic laboratory databases of medical charts in the ICU. Demographic data, comorbidities, and physical/disease status (Acute Physiology and Chronic Health Evaluation [APACHE] II score) were extracted at the time of ICU admission. Clinical symptoms, laboratory values, and sequential organ failure assessment (SOFA) scores were obtained within 24 h before catheter removal due to suspected CRBSI.

### Definitions

A suspected CRBSI was defined as the development of a new episode of fever or sepsis [5] with at least 1 additional parameter described in the 2001 International Sepsis Definitions Conference guidelines (see Additional file 5: Table S1) on a review of the information recorded in the progress notes in the medical charts [19, 20]. Fever was defined as a temperature > 38.3℃ [19]. Sepsis was defined according to the Third International Consensus Definitions for Sepsis and Septic Shock [20]. The standard protocols for antifungal and antibacterial therapy during and after catheter removal were determined empirically by the physician responsible for each patient based on available microbial culture results.

IRINC was defined as the reinsertion of a new catheter at a new site for continuous treatment within 24 h after prompt CVC removal [10, 11]. In this study, IRINC was defined as reinsertion within 24 h as opposed to delayed or no reinsertion and was also considered a key management strategy for CVC with suspected CRBSI [10]. The CVC was promptly removed, and after 24 h of watchful waiting, microbiological culture results were available. Patients were defined as not undergoing IRINC if the CVC was reinserted more than 24 h after removal or was not reinserted at all.

CRBSI was defined according to the IDSA guidelines as catheter tip colonization with the same phenotype of microorganisms isolated from peripheral blood culture [8, 10]. Colonization of the catheter tip was defined as the presence of 15 or more colony-forming units on the tip of the CVC [5]. An earlier systematic review showed evidence supporting the use of catheter tip colonization as a surrogate end point for CRBSI [21], but catheter tip colonization does not reliably reflect treatment effects on CRBSI and is consequently more suitable for surveillance than for clinical effectiveness research [22]. Therefore, patients with CRBSI (n = 158 [73.8%]) and patients with catheter colonization (56 [26.2%]) confirmed by the microbiological test results were included in the CRBSI cohort in this study.

### Primary and secondary outcome measures

The primary outcome of this study was 30-day mortality after CVC removal in patients with suspected CRBSI. We used 30-day mortality as the primary outcome measure because previous studies showed that CRBSIs were associated with mortality, while mortality after 30 days was considered less likely to be related to CRBSI [5, 10, 23].

The secondary outcome of this study was secondary CRBSI after IRINC in patients with suspected CRBSI. CRBSI is considered the leading cause of morbidity and mortality among patients with CVCs [2, 3].

### Study sample size

Given that this study was a hypothesis-driven exploratory study, no attempt was made to estimate the necessary sample size for the study. Instead, all eligible patients in the research unit were enrolled to achieve the maximum statistical power.

### Statistical analysis

Propensity score matching was used to balance the differences in baseline characteristics between patients who underwent IRINC and those who did not. A propensity score, the probability of undergoing IRINC, was estimated using logistic regression based on ICU admission demographics, comorbidity, physical/disease status, suspected infection period CVC information, clinical symptoms, concurrent medication use, intervention, disease status, and laboratory results. Propensity score matching was implemented using a nearest-neighbour strategy with a minimum caliper of 0.1 [24]. The caliper for the matching was specified in the nearest-neighbour strategy if the unspecified approach did not result in satisfactory balance [25], or inverse probability weighting was adopted to account for potential imbalanced factors [26]. The ratio was one patient receiving no IRINC to one matched patient receiving IRINC. The standardized mean difference (SMD) was used to assess the balance of baseline covariates between the non-IRINC and IRINC groups in the matched cohort. An SMD of less than 0.10 indicated a good balance [24].

The 30-day mortality was evaluated using Kaplan–Meier curve analysis and compared with a log-rank test. To further validate the impacts of IRINC on 30-day mortality, Cox proportional hazards regression was used to compare the mortality of patients undergoing IRINC and those not undergoing IRINC in a propensity score-matched cohort, with robust sandwich estimates to account for the clustering within matched sets [27]. The proportional hazards assumption was tested on the basis of Schoenfeld residuals [28]. Finally, as a component of the primary outcome, we assessed the association of IRINC with subsequent mortality among patients in subgroups with and without CRBSI by building new propensity scores and Cox models. In addition, to validate the results of the propensity score matched cohort, we used a competing risk survival model to identify risk factors for the development of a secondary CRBSI (primary event) in the propensity score-matched cohort, and for patients without secondary CRBSI, death and discharge were treated as competing events in the competing risk survival model [4].

## Results

### Study patients

**Fig. 1:**
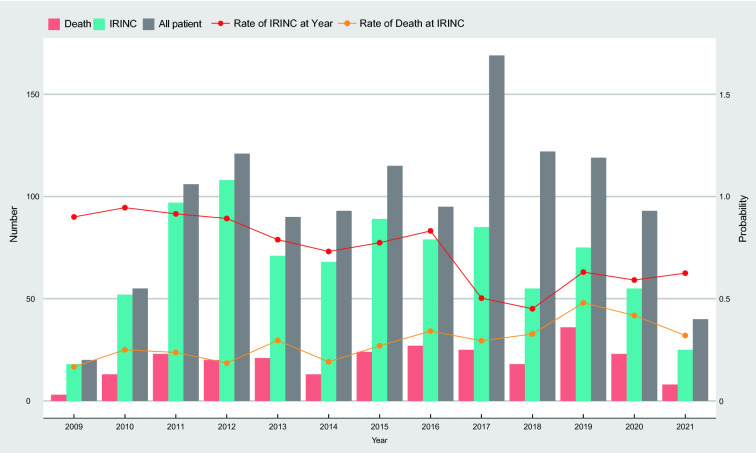
Patient Distribution of the Study by Year, According to Number of Immediate Reinsertions of New Catheters (IRINCs) and Death. The red curve shows the percentage of IRINCs prompted by suspected infections. The yellow curve shows the percentage of IRINC recipients who died

A total of 1238 patients with CVC removal due to suspected CRBSI were identified, including 877 (70.8%) patients who underwent IRINC and 361 (29.2%) patients who did not undergo IRINC from January 2009 through April 2021 (Fig. 1). The mean age of the patients was 61.3 years (SD, 17.4), and 885 patients (71.5%) were men. Table 1 shows the demographic and clinical characteristics of the study patients before and after propensity score matching. Patients who underwent IRINC had higher disease severity on admission, more severe clinical symptoms (fever, shock), more confirmed CRBSI, and more severe organ failure (SOFA score, laboratory results) than those who did not undergo IRINC. The covariates were well balanced in the propensity score-matched cohort, with all SMDs less than 10% (for the propensity score, see Additional file 1: Figure S1).

**Table 1:** Baseline Characteristics of Patients Who Underwent Central Venous Catheter removal for Suspected Infection, According to IRINC or not, before and after Propensity-Score-Matched

Characteristic	Before Propensity Score Matching			After Propensity Score Matching		
	No IRINC (n = 361)	IRINC (n = 877)	SMD (%)	No IRINC (n = 341)	IRINC (n = 341)	SMD (%)
Admission						
Female	112 (31.0)	241 (27.5)	7.8	102 (29.9)	96 (28.2)	3.9
Age, mean (SD)	59.34 (17.70)	62.11 (17.18)	15.9	59.91 (17.68)	59.57 (17.52)	2.0
Diagnostic			12.1			6.5
Medical	211 (58.4)	546 (62.3)		202 (59.2)	191 (56.0)	
Surgical	106 (29.4)	211 (24.1)		96 (28.2)	104 (30.5)	
Traumatology	44 (12.2)	120 (13.7)		43 (12.6)	46 (13.5)	
APACHE II score, mean (SD)	18.58 (6.79)	20.88 (7.18)	32.9	18.91 (6.71)	19.28 (6.46)	5.7
Chronic comorbidity						
Chronic Obstructive Pulmonary Disease	28 (7.8)	102 (11.6)	13.1	28 (8.2)	26 (7.6)	2.2
Diabetes Mellitus	67 (18.6)	161 (18.4)	0.5	62 (18.2)	56 (16.4)	4.7
Malignancy	36 (10.0)	89 (10.1)	0.6	34 (10.0)	38 (11.1)	3.8
Renal insufficiency	69 (19.1)	143 (16.3)	7.4	63 (18.5)	66 (19.4)	2.2
CVC removed day with suspected CRBSI						
Treatment Interventions						
Vasoconstrictor Agents	167 (46.3)	481 (54.8)	17.2	159 (46.6)	168 (49.3)	5.3
Corticosteroids	66 (18.3)	149 (17.0)	3.4	63 (18.5)	56 (16.4)	5.4
Anticoagulant	106 (29.4)	291 (33.2)	8.2	99 (29.0)	97 (28.4)	1.3
Antibiotics	252 (69.8)	658 (75.0)	11.7	237 (69.5)	232 (68.0)	3.2
Renal replacement therapy	122 (33.8)	280 (31.9)	4.0	115 (33.7)	108 (31.7)	4.4
Mechanical ventilation	217 (60.1)	559 (63.7)	7.5	205 (60.1)	201 (58.9)	2.4
Catheter information						
Site			16.8			4.3
Jugular	203 (56.2)	510 (58.2)		193 (56.6)	188 (55.1)	
Subclavian	79 (21.9)	229 (26.1)		78 (22.9)	77 (22.6)	
Femoral	79 (21.9)	138 (15.7)		70 (20.5)	76 (22.3)	
Catheter-days, days	9.06 (4.92)	9.45 (4.93)	8.0	9.13 (4.95)	9.60 (5.13)	9.4
Clinical symptoms and status						
Temperature, mean (SD)	38.22 (0.84)	38.38 (0.81)	19.2	38.24 (0.84)	38.19 (0.82)	6.0
Mean Arterial Pressure, mean (SD)	81.70 (17.99)	77.63 (16.20)	23.8	81.10 (17.51)	80.52 (16.83)	3.4
SOFA, mean (SD)	10.17 (3.38)	10.99 (3.80)	23.0	10.23 (3.40)	10.16 (3.62)	2.1
Acute Respiratory Distress Syndrome	64 (17.7)	122 (13.9)	10.5	56 (16.4)	60 (17.6)	3.1
Catheter-related bloodstream infections	46 (12.7)	168 (19.2)	17.6	45 (13.2)	40 (11.7)	4.4
Laboratory results, mean (SD)						
Blood glucose level, mmol/L	9.93 (3.74)	10.42 (3.79)	13.1	10.00 (3.79)	9.76 (3.49)	6.7
Leukocyte Count, × 10^9^/L	13.92 (7.25)	14.86 (8.31)	12.0	14.07 (7.32)	13.96 (7.18)	1.5
Neutrophils, × 10^9^/L	11.68 (7.51)	12.49 (7.87)	10.5	11.83 (7.63)	11.52 (6.06)	4.5
Plasma procalcitonin, ug/L	6.24 (44.49)	6.03 (14.40)	0.6	4.08 (11.30)	5.08 (13.00)	8.2
Creatinine, µmol/L	145.73 (147.18)	146.57 (135.23)	0.6	147.58 (148.19)	139.71 (139.22)	5.5
Activated Partial Thromboplastin Time, seconds	41.60 (15.66)	40.15 (20.73)	7.9	41.77 (16.05)	41.25 (22.07)	2.7
International Normalized Ratio	1.26 (1.00)	1.34 (0.76)	8.6	1.27 (1.03)	1.34 (0.80)	6.9
Platelet, × 10^9^/L	203.83 (144.12)	184.36 (120.54)	14.7	199.67 (143.41)	195.58 (118.79)	3.1
Plasma total bilirubin, μmol/L	34.82 (66.65)	38.39 (63.39)	5.5	33.59 (64.13)	32.09 (50.53)	2.6
PaO_2_, mmHg	113.40 (36.44)	109.05 (38.17)	11.6	112.87 (36.82)	113.55 (42.18)	1.7
Lactic acid, mmol/L	2.05 (2.25)	2.19 (2.91)	5.4	2.06 (2.31)	2.04 (2.98)	1.0

IRNIC, Immediate Reinsertion of New Catheters; SD: Standard Deviation; SMD, Standardized Mean Difference; APACHE, Acute Physiology and Chronic Health Evaluation; SOFA, Sepsis-related Organ Failure Assessment; PaO_2_, Partial Pressure of Oxygen

### Primary outcome

**Fig. 2:**
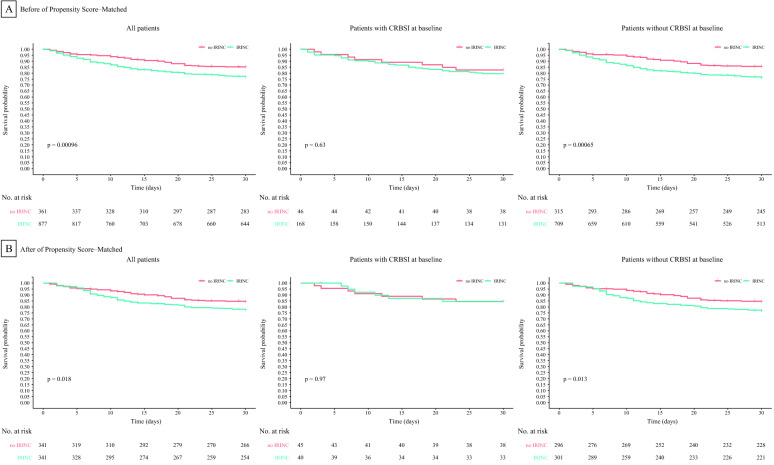
Cumulative Risks of 30-day Mortality Before and After Propensity Score-Matching. All reported P values were obtained from the log-rank test. Kaplan–Meier curve analysis, according to IRINC or no IRINC, with subgroups defined by CRBSI or no CRBSI before and after propensity score matching

**Fig. 3:**
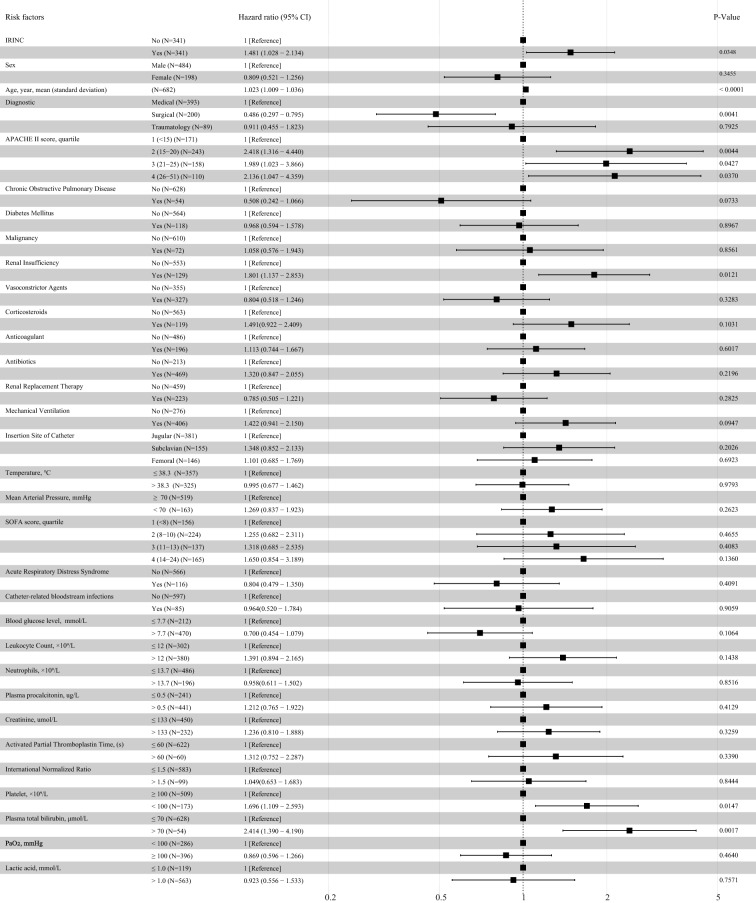
Multivariate Cox Proportional Hazards Regression Was Used to Estimate the 30-day Mortality Risk in a Propensity Score-Matched Cohort. APACHE, Acute Physiology and Chronic Health Evaluation; SOFA, Sequential Organ Failure Assessment

In the 682-patient propensity score-matched cohort, 76 deaths (22.3%) occurred in the IRINC group, and 51 (15.0%) occurred in the non-IRINC group. In Kaplan–Meier analysis, IRINC was also associated with an increased risk of 30-day mortality before matching (*P* = 0.00096 by the log-rank test, HR 1.662, 95% CI 1.225 to 2.254) and after matching (*P* = 0.018 by the log-rank test, HR 1.532, 95% CI 1.075 to 2.185) (Fig. 2). Figure 3 shows that after multivariable, multilevel adjustment, IRINC was associated with an increased risk of 30-day mortality (76/341 [22.3%] vs. 51/341 [15.0%], HR, 1.481; 95% CI, 1.028 to 2.134), which corresponded to a 48.1% higher risk of death from IRINC than not. Other risk factors for the development of 30-day mortality included an APACHE II score ≥ 16, renal insufficiency, a platelet count < 100,000 μL^−1^, and plasma total bilirubin > 70 mmol/L.

According to the results of this study (HR = 1.481), we retrospectively calculated the power value and found that it could reach 80% when the sample size of each group was 150 and 90% when the sample size of each group was 200. At present, the power has reached 99% in each group of 342, indicating that the sample size of this study is sufficient.

### Secondary outcome

For the secondary outcome, of 877 patients 30 days after IRINC, 40 patients (4.6%) experienced secondary CRBSI, 253 patients (28.8%) died without secondary CRBSI, 447 patients (51.0%) were discharged without secondary CRBSI, and 137 patients (15.6%) without secondary CRBSI did not have a known discharge location (see Additional file 5: Table S2). The competing risks analysis was similar to the results of the propensity score-matched analysis. The 30-day death risk remained higher in the IRINC group than in the non-IRINC group among patients without secondary CRBSI after adjustment for adjuvant intervention (HR, 1.52; 95% CI 1.13 to 2.04; *P* = 0.0056) (see Additional file 2: Figure S2). Finally, the attributable mortality risk of bloodstream infection in patients with suspected CRBSI was not significantly increased (HR, 1.081; 95% CI 0.964 to 1.213) over 30 days in the entire cohort based on Cox proportional hazards models (Table 2).

**Table 2:** Sensitivity and subgroup analyses of incidence and population-attributable mortality fraction of immediate reinsertion of new catheters (IRINC) in patients ^a^

Characteristic	Suspected (n = 1238)	IRINC (n = 877)	Death (n = 202)	Day 30-Attributable Mortality Hazard Ratios (95% CI)	*P*-value
Cause ^b^					
Bloodstream Infection, n (%)	298 (24.1)	232 (26.4)	52 (25.7)	1.081 (0.964–1.213)	0.184
Pneumonia, *n* (%)	603 (48.7)	433 (49.4)	109 (54.0)	1.128 (1.031–1.233)	0.008
Other Infection, *n* (%)	337 (27.2)	212 (24.2)	41 (20.3)	1.690 (0.928–3.076)	0.086
Year ^c^					
2009–2016, *n* (%)	695 (56.1)	582 (66.4)	131 (64.9)	1.100 (1.040–1.163)	0.001
2017–2021, *n* (%)	543 (43.9)	295 (33.6)	71(35.1)	1.213 (1.049–1.402)	0.009

^a^ A population-attributable mortality fraction base on Cox proportional hazards models, which using the same covariable, we attempt to evaluate the proportion of cases of deaths that could be prevented if the influence of IRINC was removed

^**b**^ Bloodstream infection as a stratification variable including catheter-related bloodstream infection and bacteremia without catheter colonization, regardless of complications with or without other infections; Pneumonia as a stratification variable only was confirmed pneumonia in patients without combined other infections; Other infection as a stratification variable including fewer cases of infection and an unknown source of infection

^**c**^ 2009–2016 as a stratification variable because it is the rate of IRINC more than 70%, or 2017–2021 as a stratification variable because it is the rate of IRINC less than 70%, to identify the risk of attributable mortality

### Sensitivity and subgroup analyses

After propensity score matching, we found that patients with CRBSI who did not undergo IRINC were similar to those who underwent IRINC in terms of causative pathogens and disease severity (see Additional file 5: Tables S3, S4 and S5). In the IRINC group and the non-IRINC group for causative pathogens, the rates of infection with gram-negative microorganisms were 51.1% (23/45) and 37.5% (15/40) (odds ratios 1.363, 95% CI 0.834–2.228), the prevalence rates of infection with gram-positive microorganisms were 26.7% (12/45) and 35.0% (14/40) (odds ratios 0.762, 95% CI 0.401–1.449), and the prevalence rates of infection with fungal microorganisms were 24.4% (11/45) and 24.4% (11/40) (odds ratios 0.889, 95% CI 0.433–1.824), respectively. Patients in the two groups (with and without CRBSI) were classified into two subgroups according to whether IRINC was performed (IRINC and non-IRINC). In Kaplan–Meier analysis (Fig. 2), for patients with CRBSI, we did not find significant differences between the IRINC group and the non-IRINC group before matching (*P* = 0.63 by the log-rank test, HR 1.210, 95% CI 0.560 to 2.613) or after matching (*P* = 0.97 by the log-rank test, HR 0.939, 95% CI 0.368 to 2.392), and the results were consistent with Kaplan–Meier curve analysis based on new propensity scores constructed for subgroups with or without CRBSI (see Additional file 5: Tables S6 and S7, and Additional file 3: Figures S3 and Additional file 4: Figure S4). However, for patients without CRBSI (Fig. 2), IRINC was associated with an increased risk of 30-day mortality before matching (*P* = 0.00065 by the log-rank test, HR 1.768, 95% CI 1.269 to 2.464) and after matching (*P* = 0.013 by the log-rank test, HR 1.611, 95% CI 1.105 to 2.349).

## Discussion

Interestingly, we found that IRINC was associated with increased 30-day mortality in patients after prompt catheter removal due to suspected CRBSI, which was consistent across several analytic approaches and robust to multiple sensitivity analyses. This association was apparent regardless of physical/disease status, ICU admission, comorbidities, treatment interventions, clinical symptoms, disease severity with suspected infection, or causative pathogens in CRBSI. However, in subgroups of patients with CRBSI, there was no significant difference in the 30-day mortality rate between patients with and without IRINC.

The association between IRINC and an increased risk of 30-day mortality that was observed in the patient-level analysis could be due to the selection of patients or to unmeasured confounding factors, as patients who are evenly matched for the causative pathogens can still have significant adverse outcomes depending on what the offending agent is. For example, antibiotic use guided by inflammatory variables did not result in a reduced use of antibiotics compared with usual care among patients with suspected infection [29]. Some specific antibiotic agents have been found not to reduce mortality at 28 days without microbial sensitivity tests [30]. In addition, propensity score matching to balance antibiotics by use (types > 2) or not (types ≤ 2) may reduce antibiotic selection pressure without a negative impact on mortality, and an expert statement recommends de-escalation from a broad‑spectrum to a narrow‑spectrum antimicrobial [9]. Similar, perhaps, to unmeasured confounding factors is the use of vasoconstrictor agents [31], corticosteroids [32], anticoagulants [33], renal replacement therapy [34], and mechanical ventilation [35]. Finally, the competing risk analysis confirmed that the results of the propensity score-matched analysis were not subject to patient selection bias.

The decision about whether to recommend immediate or later CVC removal in this population is controversial because source control by immediate removal of suspected intravascular catheters is always urgent in patients with septic shock [5, 9, 11, 15]. Moreover, a prospective observational study conducted in 18 ICUs in Spain showed that patients with immediate CVC removal had a higher rate of 30-day mortality than those with later CVC removal in suspected CRBSI [5]. However, they did not evaluate the association of reinsertion of new catheters and mortality, which may underlie the risk of iatrogenic injuries, because rigorous quality-controlled randomized controlled trials in France showed that the rate of bloodstream infection was 0.5% to 1.2% (averaging 1.0%), the rate of mechanical complications was 0.7% to 2.1% (averaging 1.4%), and the rate of deep-vein thrombosis was 0.5% to 1.4% (averaging 0.9%), varying according to the insertion site [13]. A control study demonstrated that CVC was a more significant exposure for a composite of mortality than midline catheters [36]. We attempted to maximize the benefit to patients by considering the serious consequences of CRBSI. In reality, the evidence from our data and other previous reports has supported the idea that watchful waiting may be more reasonable than CVC removal and IRINC in patients with suspected CRBSI [5], which is a clinical practice recommended by the IDSA [10].

For patients with CRBSI in the subgroup, we did not find that mortality was higher in patients with IRINC than in those with delayed replacement or no replacement of the catheter. One can argue that the sample size of the patients with CRBSI who did not undergo IRINC was relatively small, as there were only 42 deaths, which may mean that the mortality analysis had limited power to detect a between-group difference. However, the hazard ratio that we observed was similar to that of all patients. Theoretically, CVC removal and reinsertion can remove the source of infection. In fact, CVC removal and reinsertion increase the risks of catheter recolonization and introduce other risks of complications [13, 16]. It has been reported that prompt CVC removal may not be necessary for all patients [5]. In addition, this study could not identify a certain proportion of catheter colonization after bacteraemia in all patients with CRBSI. We used bloodstream infections in general as an independent risk factor for attributed mortality risk in the subgroup and sensitivity analyses. These bloodstream infections may be associated with a good prognosis compared to other infections, and we may have overestimated the mortality risk of CRBSI among all patients with sepsis [4, 6, 7]. However, given that this study was an exploratory study based on hypotheses and that this association was apparent, we need to be cautious because of the risk of a lack of a causal relationship due to the inherent limitations of the observational study. Therefore, this association can inform future clinical trials seeking to prevent insertion complications with mortality as an end point, as they can be avoided in the great majority of cases [9] by preventing unnecessary catheter removals [5, 17] and frequent changes and reinsertion of catheters in critically ill patients.

A major limitation of the present study is our inability to explain why IRINC was associated with increased 30-day mortality based on clinical data regarding related complications. Previous studies have shown that unnecessary catheter removal is common [1, 5, 7], although routine replacement of CVCs has not been recommended in international clinical guidelines to prevent CRBSI [8, 9, 37]. A recent systematic review reported that catheter removal and reinsertion may be associated with discomfort, complication risks, and possible disruption or delay in the administration of other treatments to critically ill patients [11]. Additionally, there was an increase in the number of complications related to the insertion and use of intravascular catheters [13, 38]. Therefore, CVC insertion was a significant risk factor for a series of catheter-related complications, including high symptom burden [16, 36], secondary infection [4], severe injurious complications [13, 39], and other associated harms (e.g., air embolisms, dislodgement of thrombi, haemorrhage/bruising and arterial complications) [40–42]. Although ultrasound-guided CVC placement can easily be performed with training and it is recommended that subclavian venous catheters be inserted with ultrasound guidance to decrease the mechanical complication rate, the superiority of this strategy over another cannot be clearly demonstrated [37]. Unfortunately, our study did not provide data on the use of ultrasound guidance and related complications in each patient with IRINC. Therefore, this issue may need to be approached with caution, as unmeasured variables may have an impact on the outcome.

Another limitation of this study, as a single-centre cohort study, was that it used data covering a period of more than 12 years, and many clinical practices can possibly change in terms of care for CVC over such a long period. In addition, the exclusion of patients without catheter tip culture may mean that this cohort might not be sufficiently representative of real-world clinical practice. However, since 2009, our medical team has adhered to the guidelines that catheter cultures are not routinely obtained for all patients with CVC replacements [10]; therefore, this study population is deemed homogeneous and uniform over the study period. A homogeneous sample may be more important than representativeness, and it is possible to generalize trial results to target populations even in the absence of representative data on the target population using a single-centre study [43]. Finally, we acknowledge that the positivity assumption required for using counterfactual methods such as propensity score might not be verified in some cases. Although most of ICU patients require a CVC during their stay in the ICU, the indication for IRINC or not is complex and very dependent of the physician in charge of the patient.

## Conclusions

In this cohort study, IRINC was associated with higher 30-day mortality compared to delayed CVC or no CVC among patients with suspected CRBSI. A large-sample randomized controlled trial is needed to define the best management for CVC in cases of suspected CRBSI because IRINC may also be associated with noninfectious complications.

## Supplementary Information

**Additional file 1: Figure S1.** Propensity score matching, a plot showing covariate balance, is often constructed to demonstrate the balancing effect of matching and/or weighting. Given the same propensity score model, the matching weight method often achieves better covariate balance than matching. Abbreviations: APACHE, Acute Physiology and Chronic Health Evaluation; SOFA, Sequential Organ Failure Assessment.

**Additional file 2: Figure S2.** Competing Risk Analysis for Secondary Catheter-related Bloodstream Infection in Patients with Immediate Reinsertion of a New Catheter (IRINC) or Non-IRINC in All Patients. A competing risk analysis provides 2 measures of association: the cause-specific hazard ratio, which estimates the direct impact of the exposure of interest on the various outcomes (i.e., ICU discharge, ICU death, and the development of a secondary CRBSI), which describes the risk for the development of a secondary CRBSI while accounting for the competing events. A higher cause-specific hazard ratio for death means that there is a higher hazard for death.

**Additional file 3: Figure S3.** Multivariate Cox Proportional Hazard Regression Was Used to Estimate the 30-Day Mortality Risk in the Subgroup With CRBSI in a Propensity Score-Matched Cohort. Abbreviations: APACHE, Acute Physiology and Chronic Health Evaluation; SOFA, Sequential Organ Failure Assessment.

**Additional file 4: Figure S4.** Multivariate Cox Proportional Hazard Regression Was Used to Estimate the 30-Day Mortality Risk in the Subgroup Without CRBSI in a Propensity Score-Matched Cohort. Abbreviations: APACHE, Acute Physiology and Chronic Health Evaluation; SOFA, Sequential Organ Failure Assessment.

**Additional file 5: Table S1.** Diagnostic criteria for suspected sepsis in adults according to the 2001 International Sepsis Definitions Conference. **Table S2.** Total sample size and crude number of admissions experiencing the outcome for competing risk analysis. **Table S3.** Baseline characteristics of patients who underwent central venous catheter removal for suspected infection, according to IRINC or not, before and after propensity score matching. **Table S4.** Causative pathogens of CRBSI after suspected infection in all patients. **Table S5.** Comparison of causative pathogens between patients who underwent IRINC and those who did not undergo IRINC among patients with CRBSI after propensity score matching. **Table S6.** Baseline characteristics of patients who underwent central venous catheter removal for suspected infection, according to IRINC or not, before and after propensity score matching and inverse probability of treatment weighting in the subgroup with catheter-related bloodstream infections. **Table S7.** Baseline characteristics of patients who underwent central venous catheter removal for suspected infection, according to IRINC or not, before and after propensity score matching in the subgroup without catheter-related bloodstream infection.

## Data Availability

The datasets used and/or analysed during the current study are available from the corresponding author on reasonable request.

## Abbreviations

CVC: Central venous catheter
CRBSI: Catheter-related bloodstream infection
IRNIC: Immediate reinsertion of a new catheter
APACHE: Acute Physiology and Chronic Health Evaluation
SOFA: Sequential Organ Failure Assessment
OR: Odds ratio
CI: Confidence interval
HR: Hazard ratio, a measure of relative risk used to compare different treatments; calculated from time-to-event Cox proportional hazards regression
SD: Standard deviation
SMD: Standardized mean difference

## References

[1] Takashima M, Schults J, Mihala G, Corley A, Ullman A (2018). Complication and failures of central vascular access device in adult critical care settings. Crit Care Med.

[2] Magill SS, Edwards JR, Bamberg W, Beldavs ZG, Dumyati G, Kainer MA (2014). Multistate point-prevalence survey of health care-associated infections. N Engl J Med.

[3] Siempos II, Kopterides P, Tsangaris I, Dimopoulou I, Armaganidis AE (2009). Impact of catheter-related bloodstream infections on the mortality of critically ill patients: a meta-analysis. Crit Care Med.

[4] van Vught LA, Klouwenberg PMK, Spitoni C, Scicluna BP, Wiewel MA, Horn J (2016). Incidence, risk factors, and attributable mortality of secondary infections in the intensive care unit after admission for sepsis. JAMA.

[5] Lorente L, Martin MM, Vidal P, Rebollo S, Ostabal MI, Sole-Violan J (2014). Should central venous catheter be systematically removed in patients with suspected catheter related infection?. Crit Care.

[6] Lecronier M, Valade S, Bige N, de Prost N, Roux D, Lebeaux D (2018). Removal of totally implanted venous access ports for suspected infection in the intensive care unit: a multicenter observational study. Ann Intensive Care.

[7] Zhong Y, Zhou L, Liu X, Deng L, Wu R, Xia Z (2021). Incidence, risk factors, and attributable mortality of catheter-related bloodstream infections in the intensive care unit after suspected catheters infection: a retrospective 10-year cohort study. Infect Dis Ther.

[8] O'Grady NP, Alexander M, Burns LA, Dellinger EP, Garland J, Heard SO (2011). Guidelines for the prevention of intravascular catheter-related infections. Clin Infect Dis.

[9] Timsit JF, Ruppe E, Barbier F, Tabah A, Bassetti M (2020). Bloodstream infections in critically ill patients: an expert statement. Intensive Care Med.

[10] Mermel LA, Allon M, Bouza E, Craven DE, Flynn P, O'Grady NP (2009). Clinical practice guidelines for the diagnosis and management of intravascular catheter-related infection: 2009 update by the infectious diseases society of America. Clin Infect Dis.

[11] Janum S, Afshari A (2016). Central venous catheter (CVC) removal for patients of all ages with candidaemia. Cochrane Database Syst Rev.

[12] Gershengorn HB, Garland A, Kramer A, Scales DC, Rubenfeld G, Wunsch H (2014). Variation of arterial and central venous catheter use in United States intensive care units. Anesthesiology.

[13] Parienti JJ, Mongardon N, Megarbane B, Mira JP, Kalfon P, Gros A (2015). Intravascular complications of central venous catheterization by insertion site. N Engl J Med.

[14] Chopra V, Anand S, Hickner A, Buist M, Rogers MA, Saint S (2013). Risk of venous thromboembolism associated with peripherally inserted central catheters: a systematic review and meta-analysis. Lancet.

[15] Timsit JF, Rupp M, Bouza E, Chopra V, Karpanen T, Laupland K (2018). A state of the art review on optimal practices to prevent, recognize, and manage complications associated with intravascular devices in the critically ill. Intensive Care Med.

[16] Raad S, Chaftari AM, Hachem RY, Shah P, Natividad E, Cleeland CS (2018). Removal and insertion of central venous catheters in cancer patients is associated with high symptom burden. Expert Rev Med Devices.

[17] Rijnders BJ, Peetermans WE, Verwaest C, Wilmer A, Van Wijngaerden E (2004). Watchful waiting versus immediate catheter removal in ICU patients with suspected catheter-related infection: a randomized trial. Intensive Care Med.

[18] Sabatier C, Garcia X, Ferrer R, Duarte M, Colomina M, Alcaraz D (2015). Blood culture differential time to positivity enables safe catheter retention in suspected catheter-related bloodstream infection: a randomized controlled trial. Med Intensiva.

[19] Levy MM, Fink MP, Marshall JC, Abraham E, Angus D, Cook D (2003). 2001 SCCM/ESICM/ACCP/ATS/SIS international sepsis definitions conference. Crit Care Med.

[20] Singer M, Deutschman CS, Seymour CW, Shankar-Hari M, Annane D, Bauer M (2016). The third international consensus definitions for sepsis and septic shock (Sepsis-3). JAMA.

[21] Rijnders BJ, Van Wijngaerden E, Peetermans WE (2002). Catheter-tip colonization as a surrogate end point in clinical studies on catheter-related bloodstream infection: how strong is the evidence?. Clin Infect Dis.

[22] de Grooth HJ, Timsit JF, Mermel L, Mimoz O, Buetti N, du Cheyron D (2020). Validity of surrogate endpoints assessing central venous catheter-related infection: evidence from individual- and study-level analyses. Clin Microbiol Infect.

[23] Kahn JM, Davis BS, Yabes JG, Chang CH, Chong DH, Hershey TB (2019). Association between state-mandated protocolized sepsis care and in-hospital mortality among adults with sepsis. JAMA.

[24] Austin PC (2011). Optimal caliper widths for propensity-score matching when estimating differences in means and differences in proportions in observational studies. Pharm Stat.

[25] Reynolds HR, Adhikari S, Pulgarin C, Troxel AB, Iturrate E, Johnson SB (2020). Renin-angiotensin-aldosterone system inhibitors and risk of covid-19. N Engl J Med.

[26] Li N, Tan F, Chen W, Dai M, Wang F, Shen S (2022). One-off low-dose CT for lung cancer screening in China: a multicentre, population-based, prospective cohort study. Lancet Respir Med.

[27] Gayat E, Resche-Rigon M, Mary JY, Porcher R (2012). Propensity score applied to survival data analysis through proportional hazards models: a Monte Carlo study. Pharm Stat.

[28] Xue X, Kim MY, Gaudet MM, Park Y, Heo M, Hollenbeck AR (2013). A comparison of the polytomous logistic regression and joint cox proportional hazards models for evaluating multiple disease subtypes in prospective cohort studies. Cancer Epidemiol Biomarkers Prev.

[29] Huang DT, Yealy DM, Filbin MR, Brown AM, Chang CH, Doi Y (2018). Procalcitonin-guided use of antibiotics for lower respiratory tract infection. N Engl J Med.

[30] Dellinger RP, Bagshaw SM, Antonelli M, Foster DM, Klein DJ, Marshall JC (2018). Effect of targeted polymyxin B hemoperfusion on 28-day mortality in patients with septic shock and elevated endotoxin level: the EUPHRATES randomized clinical trial. JAMA.

[31] Hernandez G, Ospina-Tascon GA, Damiani LP, Estenssoro E, Dubin A, Hurtado J (2019). Effect of a resuscitation strategy targeting peripheral perfusion status vs serum lactate levels on 28-day mortality among patients with septic shock: the ANDROMEDA-SHOCK randomized clinical trial. JAMA.

[32] Fujii T, Luethi N, Young PJ, Frei DR, Eastwood GM, French CJ (2020). Effect of vitamin C, hydrocortisone, and thiamine vs hydrocortisone alone on time alive and free of vasopressor support among patients with septic shock: the VITAMINS randomized clinical trial. JAMA.

[33] Vincent JL, Francois B, Zabolotskikh I, Daga MK, Lascarrou JB, Kirov MY (2019). Effect of a recombinant human soluble thrombomodulin on mortality in patients with sepsis-associated coagulopathy: the SCARLET randomized clinical trial. JAMA.

[34] Barbar SD, Clere-Jehl R, Bourredjem A, Hernu R, Montini F, Bruyere R (2018). Timing of renal-replacement therapy in patients with acute kidney injury and sepsis. N Engl J Med.

[35] Wittekamp BH, Plantinga NL, Cooper BS, Lopez-Contreras J, Coll P, Mancebo J (2018). Decontamination strategies and bloodstream infections with antibiotic-resistant microorganisms in ventilated patients: a randomized clinical trial. JAMA.

[36] Mushtaq A, Navalkele B, Kaur M, Krishna A, Saleem A, Rana N (2018). Comparison of complications in midlines versus central venous catheters: are midlines safer than central venous lines?. Am J Infect Control.

[37] Timsit JF, Baleine J, Bernard L, Calvino-Gunther S, Darmon M, Dellamonica J (2020). Expert consensus-based clinical practice guidelines management of intravascular catheters in the intensive care unit. Ann Intensive Care.

[38] Hodzic S, Golic D, Smajic J, Sijercic S, Umihanic S, Umihanic S (2014). Complications related to insertion and use of Central Venous Catheters (CVC). Med Arch.

[39] Kim E, Kim BG, Lim YJ, Jeon YT, Hwang JW, Kim HC (2016). A prospective randomised trial comparing insertion success rate and incidence of catheterisation-related complications for subclavian venous catheterisation using a thin-walled introducer needle or a catheter-over-needle technique. Anaesthesia.

[40] Pinho J, Amorim JM, Araujo JM, Vilaca H, Ribeiro M, Pereira J (2016). Cerebral gas embolism associated with central venous catheter: systematic review. J Neurol Sci.

[41] Ullman AJ, Marsh N, Mihala G, Cooke M, Rickard CM (2015). Complications of central venous access devices: a systematic review. Pediatrics.

[42] Collier PE (2019). Prevention and treatment of dilator injuries during central venous catheter placement. J Vasc Surg Venous Lymphat Disord.

[43] Hong JL, Webster-Clark M, Jonsson Funk M, Sturmer T, Dempster SE, Cole SR (2019). Comparison of methods to generalize randomized clinical trial results without individual-level data for the target population. Am J Epidemiol.

