# Supplementary material for: Association of immediate reinsertion of new catheters with subsequent mortality among patients with suspected catheter infection: a cohort study
Source: Ann Intensive Care. 2022 May 7;12:38. doi: 10.1186/s13613-022-01014-8 (PMC9079203; doi:10.1186/s13613-022-01014-8)
Supplement: Supplementary file 5 — Additional file 5: Table S1. Diagnostic criteria for suspected sepsis in adults according to the 2001 International Sepsis Definitions Conference. Table S2. Total sample size and crude number of admissions experiencing the outcome for competing risk analysis. Table S3. Baseline characteristics of patients who underwent central venous catheter removal for suspected infection, according to IRINC or not, before and after propensity score matching. Table S4. Causative pathogens of CRBSI after suspected infection in all patients. Table S5. Comparison of causative pathogens between patients who underwent IRINC and those who did not undergo IRINC among patients with CRBSI after propensity score matching. Table S6. Baseline characteristics of patients who underwent central venous catheter removal for suspected infection, according to IRINC or not, before and after propensity score matching and inverse probability of treatment weighting in the subgroup with catheter-related bloodstream infections. Table S7. Baseline characteristics of patients who underwent central venous catheter removal for suspected infection, according to IRINC or not, before and after propensity score matching in the subgroup without catheter-related bloodstream infection. [file 13613_2022_1014_MOESM5_ESM.docx]

**Additional Material Table**

**Association of** **Immediate Reinsertion of New Catheters with Subsequent Mortality among Patients with Suspected Catheter Infection: A Cohort Study**

Supplemental material Content

[Table S 1. Diagnostic criteria for suspected sepsis in adults according to the 2001 International Sepsis Definitions Conference. 3](#_Toc99943531)

[Table S 2. Total sample size and crude number of admissions experiencing the outcome for competing risk analysis. 4](#_Toc99943532)

[Table S 3. Baseline characteristics of patients who underwent central venous catheter removal for suspected infection, according to IRINC or not, before and after propensity score matching. 6](#_Toc99943533)

[Table S 4. Causative pathogens of CRBSI after suspected infection in all patients*. 8](#_Toc99943534)

[Table S 5. Comparison of causative pathogens between patients who underwent IRINC and those who did not undergo IRINC among patients with CRBSI after propensity score matching.* 9](#_Toc99943535)

[Table S 6. Baseline characteristics of patients who underwent central venous catheter removal for suspected infection, according to IRINC or not, before and after propensity score matching and inverse probability of treatment weighting in the subgroup with catheter-related bloodstream infections. 10](#_Toc99943536)

[Table S 7: Baseline characteristics of patients who underwent central venous catheter removal for suspected infection, according to IRINC or not, before and after propensity score matching in the subgroup without catheter-related bloodstream infection. 12](#_Toc99943537)

# Table S 1. Diagnostic criteria for suspected sepsis in adults according to the 2001 International Sepsis Definitions Conference.

| Infection documented or suspected, and some of the following: |
| --- |
| General variables |
| Fever (core temperature >38.3°C) |
| Hypothermia (core temperature <36°C) |
| Heart rate >90 min^-1^ or >2 SD above the normal value for age |
| Tachypnoea |
| Altered mental status |
| Significant oedema or positive fluid balance (>20 mL/kg over 24 hrs) |
| Hyperglycaemia (plasma glucose >120 mg/dL or 7.7 mmol/L) in the absence of diabetes |
| Inflammatory variables |
| Leucocytosis (WBC count >12,000 μL^-1^) |
| Leucopenia (WBC count <4000 μL^-1^) |
| Normal WBC count with >10% immature forms |
| Plasma C-reactive protein >2 SD above the normal value |
| Plasma procalcitonin >2 SD above the normal value |
| Haemodynamic variables |
| Arterial hypotension (SBP >90 mmHg, MAP <70, or an SBP decrease >40 mm Hg in adults or <2 SD below normal for age) |
| mixed venous oxygen saturation >70% |
| Cardiac index >3.5 L min^-1^·M^-23^ |
| Organ dysfunction variables |
| Arterial hypoxaemia (PaO_2_/FiO_2_ <300) |
| Acute oliguria (urine output <0.5 mL·kg^-1^·hr^-1^ or 45 mmol/L for at least 2 hrs) |
| Creatinine increase >0.5 mg/dL |
| Coagulation abnormalities (INR >1.5 or APTT >60 secs) |
| Ileus (absent bowel sounds) |
| Thrombocytopaenia (platelet count <100,000 μL^-1^) |
| Hyperbilirubinaemia (plasma total bilirubin >4 mg/dL or 70 mmol/L) |
| Tissue perfusion variables |
| Hyperlactataemia (>1 mmol/L) |
| Decreased capillary refill or mottling |
| Abbreviations: WBC, white blood cell; SBP, systolic blood pressure; MAP, mean arterial blood pressure; INR, international normalized ratio; APTT, activated partial thromboplastin time. |

# Table S 2. Total sample size and crude number of admissions experiencing the outcome for competing risk analysis.

| **Risk factors** | **Total sample size** | **Total number discharged** | **Total number deceased** | **Total number with CRBSI** |
| --- | --- | --- | --- | --- |
| **Admission** | N = 1238 | N = 447 | N = 253 | N = 40 |
| APACHE 1^st^ quartile (<15) (reference) | 260 | 113 | 23 | 6 |
| APACHE 2^nd^ quartile (15-20) | 400 | 134 | 77 | 10 |
| APACHE 3^rd^ quartile (21-25) | 324 | 112 | 71 | 18 |
| APACHE 4^th^ quartile (26-51) | 257 | 88 | 82 | 6 |
| Male | 885 | 332 | 188 | 30 |
| Age (per 1 year) | 1238 | 447 | 253 | 40 |
| Medical admission | 757 | 255 | 188 | 25 |
| Surgical admission | 317 | 135 | 44 | 9 |
| Traumatology admission | 164 | 57 | 21 | 6 |
| SOFA 1^st^ quartile (<8) (reference) | 234 | 94 | 23 | 9 |
| SOFA 2^nd^ quartile (8-10) | 398 | 159 | 60 | 11 |
| SOFA 3^rd^ quartile (11-13) | 237 | 79 | 50 | 8 |
| SOFA 4^th^ quartile (14-24) | 369 | 115 | 120 | 12 |
| Acute respiratory distress syndrome | 186 | 56 | 41 | 5 |
| **Chronic comorbidity** |  |  |  |  |
| Chronic obstructive pulmonary disease | 130 | 62 | 31 | 4 |
| Diabetes mellitus | 228 | 70 | 58 | 9 |
| Malignancy | 125 | 43 | 33 | 1 |
| Renal insufficiency | 212 | 64 | 67 | 2 |
| **CVC removal day with suspected CRBSI** |  |  |  |  |
| **Interventions on suspected infection** |  |  |  |  |
| Vasoconstrictive agents | 648 | 198 | 169 | 20 |
| Corticosteroids | 215 | 65 | 59 | 6 |
| Anticoagulant | 397 | 125 | 95 | 13 |
| Antibiotics | 910 | 302 | 207 | 26 |
| Tracheostomy | 410 | 167 | 66 | 16 |
| Renal replacement therapy | 402 | 139 | 103 | 6 |
| Mechanical ventilation | 776 | 265 | 180 | 20 |
| **Cause of clinical symptoms** |  |  |  |  |
| Catheter-related bloodstream infections | 207 | 83 | 41 | 13 |
| Pneumonia | 607 | 205 | 130 | 18 |
| Bacteraemia | 86 | 27 | 21 | 3 |
| Abdominal infection | 24 | 8 | 3 | 2 |
| Gastrointestinal infection | 16 | 5 | 4 | 0 |
| Soft tissue infections | 7 | 2 | 1 | 0 |
| Urinary tract infections | 40 | 18 | 10 | 1 |
| Central nervous system infections | 11 | 6 | 0 | 0 |
| Chest infection | 1 | 1 | 0 | 0 |
| Reproductive system infection | 2 | 1 | 0 | 0 |
| Biliary tract infection | 2 | 1 | 0 | 0 |
| Unknown | 235 | 90 | 43 | 3 |
| Abbreviations: APACHE, Acute Physiology and Chronic Health Evaluation; SOFA, Sequential Organ Failure Assessment. | | | | |

# Table S 3. Baseline characteristics of patients who underwent central venous catheter removal for suspected infection, according to IRINC or not, before and after propensity score matching.

| **Characteristic** | **Before Propensity Score Matching** | | | **After Propensity Score Matching** | | |
| --- | --- | --- | --- | --- | --- | --- |
|  | **No IRINC (n=361)** | **IRINC (n=877)** | **SMD (%)** | **No IRINC (n=341)** | **IRINC (n=341)** | **SMD (%)** |
| **Admission** |  |  |  |  |  |  |
| Female | 112 (31.0) | 241 (27.5) | 7.8 | 102 (29.9) | 96 (28.2) | 3.9 |
| Age, mean (SD) | 59.34 (17.70) | 62.11 (17.18) | 15.9 | 59.91 (17.68) | 59.57 (17.52) | 2.0 |
| Diagnostic |  |  | 12.1 |  |  | 6.5 |
| Medical | 211 (58.4) | 546 (62.3) |  | 202 (59.2) | 191 (56.0) |  |
| Surgical | 106 (29.4) | 211 (24.1) |  | 96 (28.2) | 104 (30.5) |  |
| Traumatology | 44 (12.2) | 120 (13.7) |  | 43 (12.6) | 46 (13.5) |  |
| APACHE II score, mean (SD) | 18.58 (6.79) | 20.88 (7.18) | 32.9 | 18.91 (6.71) | 19.28 (6.46) | 5.7 |
| **Chronic comorbidity** |  |  |  |  |  |  |
| Chronic obstructive pulmonary disease | 28 (7.8) | 102 (11.6) | 13.1 | 28 (8.2) | 26 (7.6) | 2.2 |
| Diabetes mellitus | 67 (18.6) | 161 (18.4) | 0.5 | 62 (18.2) | 56 (16.4) | 4.7 |
| Malignancy | 36 (10.0) | 89 (10.1) | 0.6 | 34 (10.0) | 38 (11.1) | 3.8 |
| Renal insufficiency | 69 (19.1) | 143 (16.3) | 7.4 | 63 (18.5) | 66 (19.4) | 2.2 |
| **CVC removal day with suspected CRBSI** |  |  |  |  |  |  |
| **Treatment Interventions** |  |  |  |  |  |  |
| Vasoconstrictive agents | 167 (46.3) | 481 (54.8) | 17.2 | 159 (46.6) | 168 (49.3) | 5.3 |
| Corticosteroids | 66 (18.3) | 149 (17.0) | 3.4 | 63 (18.5) | 56 (16.4) | 5.4 |
| Anticoagulant | 106 (29.4) | 291 (33.2) | 8.2 | 99 (29.0) | 97 (28.4) | 1.3 |
| Antibiotics | 252 (69.8) | 658 (75.0) | 11.7 | 237 (69.5) | 232 (68.0) | 3.2 |
| Renal replacement therapy | 122 (33.8) | 280 (31.9) | 4.0 | 115 (33.7) | 108 (31.7) | 4.4 |
| Mechanical ventilation | 217 (60.1) | 559 (63.7) | 7.5 | 205 (60.1) | 201 (58.9) | 2.4 |
| **Catheter information** |  |  |  |  |  |  |
| Site |  |  | 16.8 |  |  | 4.3 |
| Jugular | 203 (56.2) | 510 (58.2) |  | 193 (56.6) | 188 (55.1) |  |
| Subclavian | 79 (21.9) | 229 (26.1) |  | 78 (22.9) | 77 (22.6) |  |
| Femoral | 79 (21.9) | 138 (15.7) |  | 70 (20.5) | 76 (22.3) |  |
| Catheter-days, days | 9.06 (4.92) | 9.45 (4.93) | 8.0 | 9.13 (4.95) | 9.60 (5.13) | 9.4 |
| **Clinical symptoms and status** |  |  |  |  |  |  |
| Temperature, mean (SD) | 38.22 (0.84) | 38.38 (0.81) | 19.2 | 38.24 (0.84) | 38.19 (0.82) | 6.0 |
| Mean arterial pressure, mean (SD) | 81.70 (17.99) | 77.63 (16.20) | 23.8 | 81.10 (17.51) | 80.52 (16.83) | 3.4 |
| SOFA, mean (SD) | 10.17 (3.38) | 10.99 (3.80) | 23.0 | 10.23 (3.40) | 10.16 (3.62) | 2.1 |
| Acute respiratory distress syndrome | 64 (17.7) | 122 (13.9) | 10.5 | 56 (16.4) | 60 (17.6) | 3.1 |
| **Cause of symptomatology, n (%)** |  |  |  |  |  |  |
| Catheter-related bloodstream infections | 46 (12.7) | 168 (19.2) | 0.17.6 | 45 (13.2) | 40 (11.7) | 4.4 |
| Pneumonia | 170 (47.1) | 433 (49.4) | 0.04.6 | 162 (47.5) | 175 (51.3) | 7.6 |
| Bacteraemia | 20 (5.5) | 64 (7.3) | 0.07.2 | 18 (5.3) | 28 (8.2) | 11.7 |
| Abdominal infection | 4 (1.1) | 20 (2.3) | 0.09.1 | 3 (0.9) | 8 (2.3) | 11.7 |
| Gastrointestinal infection | 6 (1.7) | 10 (1.1) | 0.04.4 | 6 (1.8) | 3 (0.9) | 7.7 |
| Soft tissue infections | 2 (0.6) | 5 (0.6) | 0.00.2 | 1 (0.3) | 3 (0.9) | 7.7 |
| Urinary tract infections | 10 (2.8) | 29 (3.3) | 0.03.1 | 9 (2.6) | 11 (3.2) | 3.5 |
| Central nervous system infections | 2 (0.6) | 9 (1.0) | 0.05.3 | 2 (0.6) | 4 (1.2) | 6.3 |
| Chest infection | 0 (0.0) | 1 (0.1) | 0.04.8 | 0 (0.0) | 0 (0.0) | <0.1 |
| Reproductive system infection | 2 (0.6) | 0 (0.0) | 0.10.6 | 2 (0.6) | 0 (0.0) | 10.9 |
| Biliary tract infection | 0 (0.0) | 2 (0.2) | 0.06.8 | 0 (0.0) | 1 (0.3) | 7.7 |
| Unknown | 99 (27.4) | 136 (15.5) | 0.29.3 | 93 (27.3) | 68 (19.9) | 17.3 |
| **Laboratory results, mean (SD)** |  |  |  |  |  |  |
| Blood glucose level, mmol/L | 9.93 (3.74) | 10.42 (3.79) | 13.1 | 10.00 (3.79) | 9.76 (3.49) | 6.7 |
| Leucocyte count, ×10^9^/L | 13.92 (7.25) | 14.86 (8.31) | 12.0 | 14.07 (7.32) | 13.96 (7.18) | 1.5 |
| Neutrophils, ×10^9^/L | 11.68 (7.51) | 12.49 (7.87) | 10.5 | 11.83 (7.63) | 11.52 (6.06) | 4.5 |
| Plasma procalcitonin, ug/L | 6.24 (44.49) | 6.03 (14.40) | 0.6 | 4.08 (11.30) | 5.08 (13.00) | 8.2 |
| Creatinine, µmol/L | 145.73 (147.18) | 146.57 (135.23) | 0.6 | 147.58 (148.19) | 139.71 (139.22) | 5.5 |
| Activated partial thromboplastin time, seconds | 41.60 (15.66) | 40.15 (20.73) | 7.9 | 41.77 (16.05) | 41.25 (22.07) | 2.7 |
| International normalized ratio | 1.26 (1.00) | 1.34 (0.76) | 8.6 | 1.27 (1.03) | 1.34 (0.80) | 6.9 |
| Platelet, ×10^9^/L | 203.83 (144.12) | 184.36 (120.54) | 14.7 | 199.67 (143.41) | 195.58 (118.79) | 3.1 |
| Plasma total bilirubin, μmol/L | 34.82 (66.65) | 38.39 (63.39) | 5.5 | 33.59 (64.13) | 32.09 (50.53) | 2.6 |
| PaO_2_, mmHg | 113.40 (36.44) | 109.05 (38.17) | 11.6 | 112.87 (36.82) | 113.55 (42.18) | 1.7 |
| Lactic acid, mmol/L | 2.05 (2.25) | 2.19 (2.91) | 5.4 | 2.06 (2.31) | 2.04 (2.98) | 1.0 |
| Abbreviation: IRNIC, Immediate Reinsertion of New Catheters; SD: Standard Deviation; SMD, Standardized Mean Difference; APACHE, Acute Physiology and Chronic Health Evaluation; SOFA, Sequential Organ Failure Assessment; PaO_2_, Partial Pressure of Oxygen. | | | | | | |

# Table S 4. Causative pathogens of CRBSI after suspected infection in all patients*.

| **Gram-negative bacteria** | **Number** | **Percentage*** | **Gram-positive bacteria** | **Number** | **Percentage*** | **Fungi** | **Number** | **Percentage*** |
| --- | --- | --- | --- | --- | --- | --- | --- | --- |
|  | 109 | 50.93 |  | 55 | 25.70 |  | 54 | 25.23 |
| *Acinetobacter baumannii* | 65 | 30.37 | *Staphylococcus haemolyticus* | 18 | 8.41 | *Candida albicans* | 25 | 11.68 |
| *Burkholderia cepacia* | 1 | 0.47 | *Staphylococcus epidermidis* | 17 | 7.94 | *Candida glabrata* | 8 | 3.74 |
| *Stenothrophomas maltophilia* | 3 | 1.40 | *Enterococcus faecium* | 8 | 3.74 | *Candida parapsilosis* | 6 | 2.80 |
| *Corynebacterium germannii* | 1 | 0.47 | *Staphylococcus aureus* | 6 | 2.80 | *candida tropicalis* | 5 | 2.34 |
| *Enterobacter cloacae* | 5 | 2.34 | *Staphylococcus capitis* | 2 | 0.93 | *Candida haemulonii* | 2 | 0.93 |
| *Enterobacteriaceae* | 1 | 0.47 | *Coagulase negative staphylococcus* | 2 | 0.93 | *Candida krusei* | 2 | 0.93 |
| *Escherichia coli* | 4 | 1.87 | *staphylococcus caprae* | 1 | 0.47 | *Simulon Candida* | 2 | 0.93 |
| *Klebsiella pneumoniae* | 12 | 5.61 | *Staphylococcus hominis* | 1 | 0.47 | *Candida oleophila* | 1 | 0.47 |
| *Proteus mirabilis* | 2 | 0.93 |  |  |  | *Candida croxa* | 1 | 0.47 |
| *Pseudomonas aeruginosa* | 6 | 2.80 |  |  |  | *Candida famata* | 1 | 0.47 |
| *Pseudomonas putida* | 2 | 0.93 |  |  |  | *Candida parapsilosis* | 1 | 0.47 |
| *Rolstonia mannitol* | 1 | 0.47 |  |  |  |  |  |  |
| *Serratia marcescens* | 1 | 0.47 |  |  |  |  |  |  |
| *Cryptococcosis* | 1 | 0.47 |  |  |  |  |  |  |
| *Enterobacter aerogenes* | 1 | 0.47 |  |  |  |  |  |  |
| *nonfermentative bacilli* | 3 | 1.40 |  |  |  |  |  |  |
| *: Percentages depict the proportion of catheter tip colonization caused by the pathogen indicated. In total, 221 pathogens were assigned to 214 catheter tip colonizations; in 7 (3.3%) catheter tip colonizations, more than one pathogen was assigned as the causative factor.  Abbreviations: CRBSI: Catheter related bloodstream infection. | | | | | | | | |

# Table S 5. Comparison of causative pathogens between patients who underwent IRINC and those who did not undergo IRINC among patients with CRBSI after propensity score matching.*

|  | **No IRINC**  (n=45) | **IRINC**  (n=40) | **Odds Ratio (95% Confidence Interval) ^a^** | ***P Value*** |
| --- | --- | --- | --- | --- |
| Gram-negative bacteria, n (%) | 23 (51.1) | 15 (37.5) | 1.363 (0.834 to 2.228) | 0.275 |
| Gram-positive bacteria, n (%) | 12 (26.7) | 14 (35.0) | 0.762 (0.401 to 1.449) | 0.482 |
| Fungi, n (%) | 11 (24.4) | 11 (27.5) | 0.889 (0.433 to 1.824) | 0.807 |
| *: Percentages depict the proportion of causative pathogens species. 2 patients with catheter tip colonization had concurrent gram-positive bacteria and fungi species.  a: Causative pathogen variables were compared between the no IRINC group and IRINC group using the Pearson Chi square test.  Abbreviations: CRBSI: Catheter Related Bloodstream Infection; IRINC: Immediate Reinsertion of a New Catheter. | | | | |

# Table S 6. Baseline characteristics of patients who underwent central venous catheter removal for suspected infection, according to IRINC or not, before and after propensity score matching and inverse probability of treatment weighting in the subgroup with catheter-related bloodstream infections.

|  | **Before Propensity Score Matching** | | | **After Propensity Score Matching** | | | **After Inverse Probability of Treatment Weighting** | | |
| --- | --- | --- | --- | --- | --- | --- | --- | --- | --- |
|  | **No IRINC (n=46)** | **IRINC (n=168)** | **SMD (%)** | **No IRINC (n=36)** | **IRINC (n=36)** | **SMD (%)** | **No IRINC (n=36.3)** | **IRINC (n=36.3)** | **SMD (%)** |
| **Admission** |  |  |  |  |  |  |  |  |  |
| Sex, male | 31 (67.4) | 124 (73.8) | 14.1 | 26 (72.2) | 27 (75.0) | 6.3 | 25.8 (71.0) | 25.6 (70.7) | 0.7 |
| Age, year, mean (SD) | 58.6 (15.6) | 61.1 (17.6) | 15.0 | 58.8 (16.0) | 62.3 (16.9) | 21.3 | 58.9 (15.9) | 59.0 (17.5) | 1.1 |
| Diagnostic, n (%) |  |  | 10.9 |  |  | 18.5 |  |  | 3.7 |
| Medical | 29 (63.0) | 104 (61.9) |  | 23 (63.9) | 26 (72.2) |  | 22.0 (60.6) | 22.5 (62.2) |  |
| Surgical | 10 (21.7) | 43 (25.6) |  | 7 (19.4) | 5 (13.9) |  | 8.4 (23.1) | 7.8 (21.6) |  |
| Traumatology | 7 (15.2) | 21 (12.5) |  | 6 (16.7) | 5 (13.9) |  | 5.9 (16.3) | 5.8 (16.1) |  |
| APACHE II score, quartile, n (%) |  |  | 66.8 |  |  | 30.4 |  |  | 6.3 |
| 1^st^ quartile (<15) | 13 (28.3) | 22 (13.1) |  | 8 (22.2) | 9 (25.0) |  | 8.2 (22.6) | 9.1 (25.1) |  |
| 2^nd^ quartile (15−20) | 20 (43.5) | 47 (28.0) |  | 16 (44.4) | 12 (33.3) |  | 15.4 (42.4) | 15.0 (41.4) |  |
| 3^rd^ quartile (21−25) | 6 (13.0) | 53 (31.5) |  | 5 (13.9) | 4 (11.1) |  | 5.7 (15.7) | 5.6 (15.5) |  |
| 4^th^ quartile (26−51) | 7 (15.2) | 46 (27.4) |  | 7 (19.4) | 11 (30.6) |  | 7.0 (19.3) | 6.5 (18.0) |  |
| **Chronic comorbidity**, n (%) |  |  |  |  |  |  |  |  |  |
| Chronic obstructive pulmonary disease | 4 (8.7) | 22 (13.1) | 14.2 | 3 (8.3) | 8 (22.2) | 39.3 | 3.6 (10.0) | 4.6 (12.8) | 8.8 |
| Diabetes mellitus | 8 (17.4) | 22 (13.1) | 12. | 6 (16.7) | 5 (13.9) | 7.7 | 5.8 (16.0) | 5.4 (15.0) | 2.6 |
| Malignancy | 4 (8.7) | 14 (8.3) | 1.3 | 3 (8.3) | 1 (2.8) | 24.4 | 2.9 (8.0) | 2.4 (6.5) | 5.6 |
| Renal insufficiency | 6 (13.0) | 12 (7.1) | 19.7 | 5 (13.9) | 6 (16.7) | 7.7 | 5.1 (14.1) | 5.6 (15.4) | 3.7 |
| **CVC removal day with suspected CRBSI** |  |  |  |  |  |  |  |  |  |
| **Treatment interventions**, n (%) |  |  |  |  |  |  |  |  |  |
| Vasoconstrictive agents | 23 (50.0) | 81 (48.2) | 3.6 | 17 (47.2) | 19 (52.8) | 11.1 | 17.3 (47.8) | 17.1 (47.2) | 1.2 |
| Corticosteroids | 11 (23.9) | 28 (16.7) | 18.1 | 8 (22.2) | 8 (22.2) | <0.1 | 7.6 (20.9) | 6.9 (19.1) | 4.5 |
| Anticoagulant | 9 (19.6) | 58 (34.5) | 34.2 | 7 (19.4) | 9 (25.0) | 13.4 | 7.3 (20.1) | 7.7 (21.2) | 2.8 |
| Antibiotics | 31 (67.4) | 128 (76.2) | 19.6 | 22 (61.1) | 23 (63.9) | 5.7 | 24.0 (66.1) | 24.5 (67.8) | 3.5 |
| Renal replacement therapy | 15 (32.6) | 58 (34.5) | 4.1 | 13 (36.1) | 13 (36.1) | <0.1 | 13.4 (36.9) | 12.7 (35.0) | 4.0 |
| Mechanical ventilation | 24 (52.2) | 107 (63.7) | 23.5 | 19 (52.8) | 24 (66.7) | 28.6 | 19.9 (54.9) | 20.5 (56.6) | 3.4 |
| **Catheter information** |  |  |  |  |  |  |  |  |  |
| Insertion site of catheter |  |  | 2.9 |  |  | 8.5 |  |  | 4.0 |
| Jugular | 34 (73.9) | 122 (72.6) |  | 27 (75.0) | 28 (77.8) |  | 26.2 (72.1) | 26.5 (73.2) |  |
| Subclavian | 5 (10.9) | 19 (11.3) |  | 4 (11.1) | 4 (11.1) |  | 4.0 (11.2) | 3.6 (9.9) |  |
| Femoral | 7 (15.2) | 27 (16.1) |  | 5 (13.9) | 4 (11.1) |  | 6.1 (16.7) | 6.1 (16.9) |  |
| **Clinical symptoms and status** |  |  |  |  |  |  |  |  |  |
| Temperature, (>38.3 ℃) | 27 (58.7) | 56 (33.3) | 52.6 | 18 (50.0) | 23 (63.9) | 28.3 | 18.5 (51.1) | 18.8 (52.1) | 1.9 |
| Mean arterial pressure, (<70 mmHg) | 34 (73.9) | 109 (64.9) | 19.7 | 24 (66.7) | 21 (58.3) | 17.3 | 25.6 (70.6) | 24.8 (68.5) | 4.5 |
| SOFA score, quartile |  |  | 31.6 |  |  | 17.4 |  |  | 6.6 |
| 1^st^ quartile (<8) | 12 (26.1) | 34 (20.2) |  | 9 (25.0) | 7 (19.4) |  | 9.0 (24.8) | 9.1 (25.1) |  |
| 2^nd^ quartile (8−10) | 18 (39.1) | 53 (31.5) |  | 13 (36.1) | 15 (41.7) |  | 13.4 (37.1) | 14.1 (38.9) |  |
| 3^rd^ quartile (11−13) | 7 (15.2) | 26 (15.5) |  | 6 (16.7) | 5 (13.9) |  | 5.8 (15.9) | 4.9 (13.6) |  |
| 4^th^ quartile (14−24) | 9 (19.6) | 55 (32.7) |  | 8 (22.2) | 9 (25.0) |  | 8.0 (22.2) | 8.1 (22.5) |  |
| Acute respiratory distress syndrome | 4 (8.7) | 28 (16.7) | 24.1 | 3 (8.3) | 5 (13.9) | 17.7 | 3.1 (8.5) | 3.0 (8.4) | 0.4 |
| **Laboratory results** |  |  |  |  |  |  |  |  |  |
| Blood glucose level, (>7.7 mmol/L) | 10 (21.7) | 36 (21.4) | 0.8 | 8 (22.2) | 9 (25.0) | 6.5 | 7.1 (19.6) | 7.8 (21.6) | 5.1 |
| Leucocyte count, (>12×10⁹/L) | 20 (43.5) | 75 (44.6) | 2.3 | 17 (47.2) | 18 (50.0) | 5.6 | 15.4 (42.6) | 15.5 (43.0) | 0.8 |
| Neutrophils, (13.7×10⁹/L) | 35 (76.1) | 121 (72.0) | 9.3 | 25 (69.4) | 28 (77.8) | 19.0 | 25.8 (71.3) | 25.9 (71.6) | 0.7 |
| Plasma procalcitonin, (>0.5 ug/L) | 11 (23.9) | 51 (30.4) | 14.5 | 9 (25.0) | 9 (25.0) | <0.1 | 8.3 (23.0) | 8.0 (22.1) | 2.1 |
| Creatinine, (>133 µmol/L) | 12 (26.1) | 43 (25.6) | 1.1 | 10 (27.8) | 12 (33.3) | 12.1 | 10.5 (29.0) | 10.2 (28.1) | 2.0 |
| APTT, (>60 seconds) | 42 (91.3) | 155 (92.3) | 3.5 | 33 (91.7) | 31 (86.1) | 17.7 | 33.2 (91.6) | 32.1 (88.8) | 9.5 |
| International normalized ratio, (>1.5) | 38 (82.6) | 135 (80.4) | 5.8 | 29 (80.6) | 28 (77.8) | 6.8 | 29.8 (82.2) | 29.0 (80.2) | 5.1 |
| Platelet, (<100×10⁹/L) | 15 (32.6) | 47 (28.0) | 10.1 | 11 (30.6) | 16 (44.4) | 29.0 | 11.8 (32.5) | 12.4 (34.4) | 4.0 |
| Plasma total bilirubin, (>70 μmol/L) | 42 (91.3) | 150 (89.3) | 6.8 | 32 (88.9) | 31 (86.1) | 8.4 | 32.3 (89.0) | 32.0 (88.3) | 1.9 |
| PaO_2_, (<100 mmHg) | 26 (56.5) | 80 (47.6) | 17.9 | 20 (55.6) | 21 (58.3) | 5.6 | 19.9 (54.9) | 20.3 (56.1) | 2.5 |
| Lactic acid, (>1.0 mmol/L) | 9 (19.6) | 36 (21.4) | 4.6 | 7 (19.4) | 6 (16.7) | 7.2 | 5.7 (15.6) | 5.4 (15.0) | 1.9 |
| Abbreviation: IRNIC, Immediate Reinsertion of New Catheters; SD: Standard deviation; SMD, Standardized mean difference; APACHE, Acute Physiology and Chronic Health Evaluation; SOFA, Sequential Organ Failure Assessment; APTT: Activated partial thromboplastin time; PaO_2_, Partial pressure of oxygen. | | | | | | | | | |

# Table S 7. Baseline characteristics of patients who underwent central venous catheter removal for suspected infection, according to IRINC or not, before and after propensity score matching in the subgroup without catheter-related bloodstream infection.

| **Characteristic** | **Before Propensity Score Matching** | | | **After Propensity Score Matching** | | |
| --- | --- | --- | --- | --- | --- | --- |
|  | **No IRINC (n=315)** | **IRINC (n=709)** | **SMD (%)** | **No IRINC (n=297)** | **IRINC (n=297)** | **SMD (%)** |
| **Admission** |  |  |  |  |  |  |
| Sex, male | 218 (69.2) | 512 (72.2) | 6.6 | 204 (68.7) | 213 (71.7) | 6.6 |
| Age, year, mean (SD) | 59.45 (18.01) | 62.35 (17.08) | 16.6 | 60.52 (17.56) | 60.03 (17.23) | 2.8 |
| Diagnostic, n (%) |  |  | 15.6 |  |  | 2.4 |
| Medical | 182 (57.8) | 442 (62.3) |  | 171 (57.6) | 174 (58.6) |  |
| Surgical | 96 (30.5) | 168 (23.7) |  | 90 (30.3) | 89 (30.0) |  |
| Traumatology | 37 (11.7) | 99 (14.0) |  | 36 (12.1) | 34 (11.4) |  |
| APACHE II score, quartile, n (%) |  |  | 29.8 |  |  | 7.8 |
| 1^st^ quartile (<15) | 91 (28.9) | 134 (18.9) |  | 84 (28.3) | 76 (25.6) |  |
| 2^nd^ quartile (15−20) | 106 (33.7) | 227 (32.0) |  | 100 (33.7) | 104 (35.0) |  |
| 3^rd^ quartile (21−25) | 74 (23.5) | 188 (26.5) |  | 71 (23.9) | 69 (23.2) |  |
| 4^th^ quartile (26−51) | 44 (14.0) | 160 (22.6) |  | 42 (14.1) | 48 (16.2) |  |
| **Chronic comorbidity**, n (%) |  |  |  |  |  |  |
| Chronic obstructive pulmonary disease | 24 (7.6) | 80 (11.3) | 12.6 | 24 (8.1) | 23 (7.7) | 1.2 |
| Diabetes mellitus | 59 (18.7) | 139 (19.6) | 2.2 | 53 (17.8) | 45 (15.2) | 7.3 |
| Malignancy | 32 (10.2) | 75 (10.6) | 1.4 | 32 (10.8) | 30 (10.1) | 2.2 |
| Renal Insufficiency | 63 (20.0) | 131 (18.5) | 3.9 | 56 (18.9) | 59 (19.9) | 2.6 |
| **CVC removal day with suspected CRBSI** |  |  |  |  |  |  |
| **Treatment interventions**, number (%) |  |  |  |  |  |  |
| Vasoconstrictive agents | 144 (45.7) | 400 (56.4) | 21.5 | 141 (47.5) | 140 (47.1) | 0.7 |
| Corticosteroids | 55 (17.5) | 121 (17.1) | 1.0 | 54 (18.2) | 55 (18.5) | 0.9 |
| Anticoagulant | 97 (30.8) | 233 (32.9) | 4.4 | 95 (32.0) | 85 (28.6) | 7.3 |
| Antibiotics | 221 (70.2) | 530 (74.8) | 10.3 | 208 (70.0) | 205 (69.0) | 2.2 |
| Renal replacement therapy | 107 (34.0) | 222 (31.3) | 5.7 | 99 (33.3) | 106 (35.7) | 5.0 |
| Mechanical ventilation | 193 (61.3) | 452 (63.8) | 5.1 | 184 (62.0) | 184 (62.0) | <0.1 |
| **Catheter information** |  |  |  |  |  |  |
| Insertion site of catheter |  |  | 20.4 |  |  | 2.4 |
| Jugular | 169 (53.7) | 388 (54.7) |  | 160 (53.9) | 162 (54.5) |  |
| Subclavian | 74 (23.5) | 210 (29.6) |  | 73 (24.6) | 70 (23.6) |  |
| Femoral | 72 (22.9) | 111 (15.7) |  | 64 (21.5) | 65 (21.9) |  |
| **Clinical symptoms and status** |  |  |  |  |  |  |
| Temperature, (>38.3 ℃) | 166 (52.7) | 322 (45.4) | 14.6 | 154 (51.9) | 154 (51.9) | <0.1 |
| Mean arterial pressure, (<70 mmHg) | 72 (22.9) | 224 (31.6) | 19.7 | 68 (22.9) | 63 (21.2) | 4.1 |
| SOFA score, quartile |  |  | 16.3 |  |  | 6.5 |
| 1^st^ quartile (<8) | 64 (20.3) | 124 (17.5) |  | 58 (19.5) | 55 (18.5) |  |
| 2^nd^ quartile (8−10) | 107 (34.0) | 220 (31.0) |  | 100 (33.7) | 101 (34.0) |  |
| 3^rd^ quartile (11−13) | 66 (21.0) | 138 (19.5) |  | 63 (21.2) | 70 (23.6) |  |
| 4^th^ quartile (14−24) | 78 (24.8) | 227 (32.0) |  | 76 (25.6) | 71 (23.9) |  |
| Acute respiratory distress syndrome | 60 (19.0) | 94 (13.3) | 15.8 | 53 (17.8) | 51 (17.2) | 1.8 |
| **Laboratory results** |  |  |  |  |  |  |
| Blood glucose level, (>7.7 mmol/L) | 99 (31.4) | 196 (27.6) | 08.3 | 89 (30.0) | 90 (30.3) | 0.7 |
| Leucocyte count, (>12×10⁹/L) | 147 (46.7) | 291 (41.0) | 11.4 | 137 (46.1) | 132 (44.4) | 3.4 |
| Neutrophils, (13.7×10⁹/L) | 229 (72.7) | 473 (66.7) | 13.1 | 213 (71.7) | 212 (71.4) | 0.7 |
| Plasma procalcitonin, (>0.5 ug/L) | 121 (38.4) | 222 (31.3) | 14.9 | 113 (38.0) | 107 (36.0) | 4.2 |
| Creatinine, (>133 µmol/L) | 111 (35.2) | 269 (37.9) | 5.6 | 104 (35.0) | 111 (37.4) | 4.9 |
| Activated partial thromboplastin time, (>60 seconds) | 290 (92.1) | 650 (91.7) | 1.4 | 272 (91.6) | 273 (91.9) | 1.2 |
| International normalized ratio, (>1.5) | 282 (89.5) | 581 (81.9) | 21.8 | 265 (89.2) | 268 (90.2) | 3.3 |
| Platelet, (<100×10⁹/L) | 73 (23.2) | 207 (29.2) | 13.7 | 70 (23.6) | 66 (22.2) | 3.2 |
| Plasma total bilirubin, (>70 μmol/L) | 289 (91.7) | 625 (88.2) | 12.0 | 271 (91.2) | 269 (90.6) | 2.3 |
| PaO_2_, (<100 mmHg) | 192 (61.0) | 373 (52.6) | 16.9 | 180 (60.6) | 179 (60.3) | 0.7 |
| Lactic acid, (>1.0 mmol/L) | 41 (13.0) | 123 (17.3) | 12.1 | 40 (13.5) | 39 (13.1) | 1.0 |
| Abbreviation: IRNIC, Immediate Reinsertion of New Catheters; SD: Standard deviation; SMD, Standardized mean difference; APACHE, Acute Physiology and Chronic Health Evaluation; SOFA, Sequential Organ Failure Assessment; PaO_2_, Partial pressure of oxygen. | | | | | | |
